# Comprehensive genomic diagnosis of non-syndromic and syndromic hereditary hearing loss in Spanish patients

**DOI:** 10.1186/s12920-018-0375-5

**Published:** 2018-07-09

**Authors:** Rubén Cabanillas, Marta Diñeiro, Guadalupe A. Cifuentes, David Castillo, Patricia C. Pruneda, Rebeca Álvarez, Noelia Sánchez-Durán, Raquel Capín, Ana Plasencia, Mónica Viejo-Díaz, Noelia García-González, Inés Hernando, José L. Llorente, Alfredo Repáraz-Andrade, Cristina Torreira-Banzas, Jordi Rosell, Nancy Govea, Justo Ramón Gómez-Martínez, Faustino Núñez-Batalla, José A. Garrote, Ángel Mazón-Gutiérrez, María Costales, María Isidoro-García, Belén García-Berrocal, Gonzalo R. Ordóñez, Juan Cadiñanos

**Affiliations:** 1 Instituto de Medicina Oncológica y Molecular de Asturias (IMOMA) S. A, Avda. Richard Grandío s/n, 33193 Oviedo, Spain; 2 Disease Research And Medicine (DREAMgenics) S. L., Oviedo, Spain; 3 Hospital Universitario Central de Asturias, Oviedo, Spain; 4 Hospital Álvaro Cunqueiro, Vigo, Spain; 5 Hospital Universitario Son Espases, Palma de Mallorca, Spain; 6 Hospital Universitario Río Hortega, Valladolid, Spain; 7 Hospital Universitario Marqués de Valdecilla, Santander, Spain; 8 Instituto de Investigación Biomédica de Salamanca, Salamanca, Spain

**Keywords:** Hereditary, Hearing loss, Precision, Diagnostics, NGS, Gene panel

## Abstract

**Background:**

Sensorineural hearing loss (SNHL) is the most common sensory impairment. Comprehensive next-generation sequencing (NGS) has become the standard for the etiological diagnosis of early-onset SNHL. However, accurate selection of target genomic regions (gene panel/exome/genome), analytical performance and variant interpretation remain relevant difficulties for its clinical implementation.

**Methods:**

We developed a novel NGS panel with 199 genes associated with non-syndromic and/or syndromic SNHL. We evaluated the analytical sensitivity and specificity of the panel on 1624 known single nucleotide variants (SNVs) and indels on a mixture of genomic DNA from 10 previously characterized lymphoblastoid cell lines, and analyzed 50 Spanish patients with presumed hereditary SNHL not caused by *GJB2/GJB6*, *OTOF* nor *MT-RNR1* mutations.

**Results:**

The analytical sensitivity of the test to detect SNVs and indels on the DNA mixture from the cell lines was > 99.5%, with a specificity > 99.9%. The diagnostic yield on the SNHL patients was 42% (21/50): 47.6% (10/21) with autosomal recessive inheritance pattern (*BSND*, *CDH23*, *MYO15A*, *STRC* [*n* = 2], *USH2A* [*n* = 3], *RDX*, *SLC26A4*); 38.1% (8/21) autosomal dominant (*ACTG1* [n = 3; 2 de novo], *CHD7*, *GATA3* [de novo], *MITF*, *P2RX2, SOX10*), and 14.3% (3/21) X-linked (*COL4A5* [de novo], *POU3F4*, *PRPS1*). 46.9% of causative variants (15/32) were not in the databases. 28.6% of genetically diagnosed cases (6/21) had previously undetected syndromes (Barakat, Usher type 2A [n = 3] and Waardenburg [n = 2]). 19% of genetic diagnoses (4/21) were attributable to large deletions/duplications (*STRC* deletion [n = 2]; partial *CDH23* duplication; *RDX* exon 2 deletion).

**Conclusions:**

In the era of precision medicine, obtaining an etiologic diagnosis of SNHL is imperative. Here, we contribute to show that, with the right methodology, NGS can be transferred to the clinical practice, boosting the yield of SNHL genetic diagnosis to 50–60% (including *GJB2*/*GJB6* alterations), improving diagnostic/prognostic accuracy, refining genetic and reproductive counseling and revealing clinically relevant undiagnosed syndromes.

**Electronic supplementary material:**

The online version of this article (10.1186/s12920-018-0375-5) contains supplementary material, which is available to authorized users.

## Background

Congenital profound deafness affects ~ 1 in 1000 live births and an additional 1 in 1000 children will suffer from hearing loss (HL) before becoming adult [1]. Up to 60% of congenital/early-onset sensorineural HL (SNHL) is caused by genetic factors and often appears in the absence of a family history for deafness. Although alterations in the *GJB2* and *GJB6* genes *(DNFB1* locus) account for a large proportion of cases in different populations (10–40%) [2, 3], many cases remain undiagnosed after *GJB2/GJB6* testing. This is not surprising given the extreme genetic and phenotypic heterogeneity of HL, with more than 400 syndromes that include HL as a feature and more than 100 genes associated with nonsyndromic SNHL [1]. With next-generation sequencing (NGS) technology, it has become feasible and affordable to routinely sequence a large number of genes per patient [4]. Therefore, genetic diagnosis of SNHL has evolved from single-mutation Sanger sequencing to comprehensive multi-gene testing, and NGS has become the new standard of care [5].

Accordingly, once a case of newborn SNHL is confirmed, testing for congenital cytomegalovirus infection and NGS are recommended [6]. Obtaining a SNHL genetic diagnosis has a number of advantages for patients and parents [7]: it provides information about genetic heritability; it helps diagnosing or excluding syndromic causes of HL to better define medical and educational needs; it can also provide information about the evolution of the HL and/or of its associated syndromic features, improving prognostic accuracy; and it can prevent other unnecessary and costly testing [5]. Furthermore, a genetic diagnosis can contribute to prevent triggers such as aminoglycosides in mitochondrial mutation carriers [8], or even to improve treatment selection, including future mutation-driven clinical trials [9, 10].

In order to implement targeted NGS into the clinical practice, there is an urgent need to solve a number of issues such as the selection of the most efficient gene panel, the achievement of high analytical specificity and sensitivity, and the establishment of pipelines able to unambiguously define the clinical impact of genetic variants [11]. Therefore, the aims of this study are: (1) to present the development and validation of a NGS-based approach for the genetic diagnosis of patients with hereditary syndromic and non-syndromic SNHL; (2) to pinpoint and resolve the main problems associated with the introduction of targeted NGS into routine deafness diagnostics; (3) to evaluate the panel’s performance and diagnostic yield; and (4) to initiate a comprehensive catalogue of the Spanish genome-wide SNHL variation spectrum.

## Methods

### Purpose of test

The aim of the performed test (OTOgenics™) was to detect the molecular basis of individual clinical diagnoses of sensorineural or mixed hearing loss after non-genetic causes had been explored and not identified.

### Design of panel content: Rationale for inclusion of specific genes

Genes associated with prelingual, postlingual and adult-onset sensorineural or mixed HL, either symmetric or asymmetric, irrespective of the pattern of inheritance, and including both syndromic and non-syndromic forms, were considered. To generate a preliminary gene list, the professional version of the Human Gene Mutation Database (HGMD) was queried to identify genes associated to HL, using as search keywords a list of phenotypes potentially related to hearing defects (Additional file 1). The resulting gene list was manually curated by analysis of the literature and information available in the databases (HGMD, OMIM, PubMed, GeneReviews, and the Hereditary Hearing Loss Homepage; last accessed 19/09/2017) to identify those fulfilling the following criteria: I) the gene had been associated to sensorineural and/or mixed HL phenotypes (as opposed to exclusively conductive HL), II) there existed published evidence supporting the gene-phenotype association in at least two independent families and III) at least one of the existing publications demonstrated convincing cosegregation of phenotype with gene variants. Based on the curation results, a tiered classification system was devised as previously proposed by Abou Tayoun et al. [11]. Genes with strong/moderate association with HL (fulfilling criteria I, II and III described above, and corresponding mainly to Evidence level 3 according to Abou Tayoun et al. [11]) formed tier 1, while genes with weak/preliminary association (fulfilling criterion I, but not criteria II and/or III, and corresponding mainly to Evidence level 2 according to Abou Tayoun et al. [11]) were grouped in tier 2. The panel evolved with revision of newly published literature, yielding versions v1, v2 and v3 (Additional file 2, Additional file 3 and Table 1, respectively). v1–2 were used during the research and development phase of the study, whereas v3 was considered the first clinical-grade version of the panel. 32 cases were analyzed with v1, 13 with v2 and 5 with v3 (Additional file 4).

**Table 1:** Tier 1 and tier 2 genes included in the OTOgenics™ panel (v3)

Tier 1 (154 genes): genes with strong/moderate association with SNHL^#^											
*** ABHD12***	***BSND***	***CLRN1***	***DIABLO***	***FTO***	***HSD17B4***	***MARVELD2***	***MYO3A***	***P2RX2***	***PTPN11***	***SLC52A3***	***TMIE***
NM_001042472.2	NM_057176.2	NM_174878.2	NM_019887.5	NM_001080432.2	NM_000414.3	NM_001038603.2	NM_017433.4	NM_174873.2	NM_002834.3	NM_033409.3	NM_147196.2
*** ACTB***	***CABP2***	***COCH***	***DIAPH1***	***GATA3***	***ILDR1***	***MASP1***	***MYO6***	***PAX3***	***PTPRQ***	***SLITRK6***	***TMPRSS3***
NM_001101.3	NM_016366.2	NM_004086.2	NM_005219.4	NM_001002295.1	NM_001199799.1	NM_139125.3	NM_004999.3	NM_181457.3	NM_001145026.1	NM_032229.2	NM_024022.2
*** ACTG1***	***CACNA1D***	***COL2A1***	***DNMT1***	***GIPC3***	***KARS***	***MIR96***	***MYO7A***	***PCDH15***	***RAF1***	***SMPX***	***TPRN***
NM_001614.3	NM_000720.3	NM_001844.4	NM_001130823.1	NM_133261.2	NM_001130089.1	NR_029512.1	NM_000260.3	NM_033056.3	NM_002880.3	NM_014332.2	NM_001128228.2
*** ADGRV1***	***CCDC50***	***COL4A3***	***ECHS1***	***GJB2***	***KCNE1***	***MITF***	***MYO15A***	***PDZD7***	***RDX***	***SNAI2***	***TRIOBP***
NM_032119.3	NM_178335.2	NM_000091.4	NM_004092.3	NM_004004.5	NM_000219.5	NM_000248.3	NM_016239.3	NM_001195263.1	NM_002906.3	NM_003068.4	NM_001039141.2
*** AIFM1***	***CDH23***	***COL4A4***	***EDN3***	***GJB3***	***KCNJ10***	***MSRB3***	***NARS2***	***PEX1***	***RMND1***	***SOX10***	***TSPEAR***
NM_004208.3	NM_022124.5	NM_000092.4	NM_207034.2	NM_024009.2	NM_002241.4	NM_198080.3	NM_024678.5	NM_000466.2	NM_017909.3	NM_006941.3	NM_144991.2
*** ALMS1***	***CEACAM16***	***COL4A5***	***EDNRB***	***GJB6***	***KCNQ1***	***MT-CO1***	***NDP***	***PEX2***	***SERAC1***	***SPATA5***	***USH1C***
NM_015120.4	NM_001039213.3	NM_000495.4	NM_000115.3	NM_006783.4	NM_000218.2	NC_012920.1	NM_000266.3	NM_000318.2	NM_032861.3	NM_145207.2	NM_005709.3
*** ANKH***	***CHD7***	***COL9A1***	***EPS8L2***	***GPSM2***	***KCNQ4***	***MT-RNR1***	***NLRP3***	***PEX3***	***SERPINB6***	***STRC***	***USH1G***
NM_054027.4	NM_017780.3	NM_001851.4	NM_022772.3	NM_013296.4	NM_004700.3	NC_012920.1	NM_004895.4	NM_003630.2	NM_004568.5	NM_153700.2	NM_173477.4
*** AP1S1***	***CIB2***	***COL11A1***	***ESPN***	***GRHL2***	***LARS2***	***MT-TH***	***OPA1***	***PEX5***	***SIX1***	***SYNE4***	***USH2A***
NM_001283.3	NM_006383.3	NM_001854.3	NM_031475.2	NM_024915.3	NM_015340.3	NC_012920.1	NM_015560.2	NM_001131025.1	NM_005982.3	NM_001039876.1	NM_206933.2
*** ATP1A3***	***CISD2***	***COL11A2***	***ESRRB***	***GRXCR1***	***LHFPL5***	***MT-TK***	***OSBPL2***	***PEX6***	***SLC17A8***	***TBC1D24***	***WFS1***
NM_152296.4	NM_001008388.4	NM_080680.2	NM_004452.3	NM_001080476.2	NM_182548.3	NC_012920.1	NM_144498.2	NM_000287.3	NM_139319.2	NM_001199107.1	NM_006005.3
*** ATP6V1B1***	***CLCNKA***	***DCAF17***	***EYA1***	***HARS2***	***LHX3***	***MT-TL1***	***OTOA***	***PEX26***	***SLC19A2***	***TECTA***	***WHRN***
NM_001692.3	NM_004070.3	NM_025000.3	NM_000503.5	NM_012208.3	NM_014564.3	NC_012920.1	NM_144672.3	NM_017929.5	NM_006996.2	NM_005422.2	NM_015404.3
*** BCAP31***	***CLCNKB***	***DDX11***	***EYA4***	***HGF***	***LOXHD1***	***MT-TS1***	***OTOF***	***POU3F4***	***SLC26A4***	***TIMM8A***	***XYLT2***
NM_001139441.1	NM_000085.4	NM_030653.3	NM_004100.4	NM_000601.4	NM_144612.6	NC_012920.1	NM_194248.2	NM_000307.4	NM_000441.1	NM_004085.3	NM_022167.3
*** BCS1L***	***CLDN14***	***DFNA5***	***FGF3***	***HOXA1***	***LRP2***	***MYH9***	***OTOG***	***POU4F3***	***SLC33A1***	***TJP2***	
NM_004328.4	NM_144492.2	NM_004403.2	NM_005247.2	NM_005522.4	NM_004525.2	NM_002473.5	NM_001277269.1	NM_002700.2	NM_004733.3	NM_004817.3	
*** BRAF***	***CLPP***	***DFNB59***	***FGFR3***	***HOXB1***	***LRTOMT***	***MYH14***	***OTOGL***	***PRPS1***	***SLC52A2***	***TMC1***	
NM_004333.4	NM_006012.2	NM_001042702.3	NM_000142.4	NM_002144.3	NM_001145308.4	NM_024729.3	NM_173591.3	NM_002764.3	NM_024531.4	NM_138691.2	
Tier 2 (45 genes): genes with weak/preliminary association with SNHL^#^											
*** ADCY1***	***COL4A6***	***CRYM***	***ELMOD3***	***FGFR1***	***GTF2IRD1***	***KITLG***	***MT-CO3***	***NDUFA13***	***SIX5***	***TK2***	***TP63***
NM_021116.2	NM_001847.3	NM_001888.4	NM_032213.4	NM_023110.2	NM_016328.2	NM_000899.4	NC_012920.1	NM_015965.6	NM_175875.4	NM_004614.4	NM_003722.4
*** ATP2B2***	***COL9A2***	***DCDC2***	***EPS8***	***FGFR2***	***HMX2***	***MAF***	***MT-TA***	***NFIX***	***SLC4A11***	***TMEM132E***	
NM_001683.3	NM_001852.3	NM_016356.4	NM_004447.5	NM_000141.4	NM_005519.1	NM_005360.4	NC_012920.1	NM_001271043.2	NM_032034.3	NM_001304438.1	
*** ATP6V1B2***	***COL9A3***	***DIAPH3***	***FAM65B***	***FOXI1***	***HMX3***	***MARS2***	***MT-TE***	***PNPT1***	***SLC9A1***	***TMPRSS5***	
NM_001693.3	NM_001853.3	NM_001042517.1	NM_014722.3	NM_012188.4	NM_001105574.1	NM_138395.3	NC_012920.1	NM_033109.4	NM_003047.4	NM_030770.3	
*** BDP1***	***COQ6***	***DSPP***	***FBLN1***	***GRXCR2***	***HOMER2***	***MCM2***	***MT-TS2***	***SEMA3E***	***SLC26A5***	***TNC***	
NM_018429.2	NM_182476.2	NM_014208.3	NM_006486.2	NM_001080516.1	NM_004839.3	NM_004526.3	NC_012920.1	NM_012431.2	NM_198999.2	NM_002160.3	

^#^Sensorineural hearing loss

### Sample types

4 ml of peripheral blood in conventional EDTA-tubes or ≥ 200 ng of germline genomic DNA (quantitated by a fluorimetric method) were required per patient.

### Sample preparation and evaluation of genomic DNA integrity

Germline genomic DNA was isolated as previously described [12] and calculation of its DNA integrity number (DIN) was performed using the Genomic DNA ScreenTape Assay on a TapeStation 4200 system (Agilent Technologies, Santa Clara, CA, USA), following the manufacturer’s instructions.

### Library preparation, target enrichment and sequencing

Library preparation was performed on genomic DNA physically sheared by ultrasonication on a Covaris S2 instrument (Covaris, MA, USA). For library construction and gene target enrichment by hybrid capture, the SureSelectXT protocol was followed, as previously described [12]. This approach has a series of advantages over other library construction and target enrichment methods. Thus, libraries from randomly fragmented DNA show higher complexities than PCR-based ones, enabling the identification and removal of PCR duplicates (important for the accurate identification of low frequency variants present in mosaic patients) [13]. Additionally, capture probes, although laborious to use, are more tolerant to mismatches than PCR primers, circumventing issues of allelic dropout (underrepresentation or absence of an existing allele in the library) caused by polymorphisms in the hybridization sequence that can be observed in amplification-based assays [13]. Finally, capturing and sequencing at least all coding exons of every targeted gene, and not just hotspots, facilitates creating background references for CNV calling. Sequencing was performed on a NextSeq500 sequencer (Illumina, CA, USA), following the manufacturers specifications. The optimized NGS diagnostic pipeline (OTOgenics™) targets the coding exons and intron-exon junctions of 199 genes (v3) (Table 1).

### Bioinformatics for variant identification and annotation

NGS results were processed using the bioinformatics software HD Genome One (DREAMgenics, Oviedo, Spain), certified with IVD/CE-marking. The pipeline has been adapted from the one previously described as part of the ONCOgenics NGS platform [12], the performance of which has been externally evaluated through participation in the Oncogene Panel Testing schemes organized by the European Molecular Genetics Quality Network (EMQN), obtaining satisfactory results (maximum genotyping score) for three consecutive years (2015, 2016 and 2017). The analysis workflow was at follows:

### FASTQ read generation, alignment and duplicate removal

FASTQ reads were generated from base call files (BCL) produced by the Illumina NextSeq500 sequencing platform using the bcl2fastq2 v2.19 Conversion Software (https://support.illumina.com/sequencing/sequencing_software/bcl2fastq-conversion-software.html). Raw FASTQ files were evaluated using quality control checks from FastQC (http://www.bioinformatics.babraham.ac.uk/projects/fastqc/) and Trimmomatic was employed to remove low quality bases, adapters and other technical sequences. Each FASTQ file was aligned to the human reference genome (GRCh37/hg19 before 2017; GRCh38/hg38 afterwards) using BWA-mem [14] generating sorted BAM files with SAMtools [15]. Reads from the same libraries were then merged and optical and PCR duplicates were removed using Picard (http://broadinstitute.github.io/picard/).

### SNV/Indel identification

SNVs and indels were identified using a variation of Sidrón algorithm, previously described [16], with the following parameters: total read depth ≥ 6, mutated allele count ≥3, variant frequency ≥ 0.1, base quality ≥20, mapping quality ≥30. Stricter criteria (total read depth ≥ 10, mutated allele count ≥4) were applied before the selection of reportable variants. Manual inspection was then carried out to discard false positives and avoid missing true variants not meeting those criteria (i.e long indels with underestimated frequencies).

### CNV identification

The detection of CNVs was performed with an adapted version of the exome2cnv algorithm, incorporating a combination of read depth and allelic imbalance computations for copy number assessment. The algorithm employs a background of pooled samples processed using the same capturing protocol and sequencing technology [12, 17]. For increased sensitivity in the detection of large homozygous deletions, genomic regions with no sequencing coverage in an individual sample, but showing proper coverages in the remaining samples, were identified.

### Variant annotation

Variants were annotated using several databases containing functional (Ensembl, CCDS, RefSeq, Pfam), populational (dbSNP, 1000 Genomes, ESP6500, ExAC) and disease-related (Clinvar, HGMD professional) information, as well as 12 scores from algorithms for prediction of the impact caused by nonsynonymous variants on the structure and function of the protein (SIFT [18], PolyPhen2 [19], PROVEAN [20], Mutation Assessor [21], Mutation Taster [22], LRT [23], MetaLR, MetaSVM [24], FATHMM, FATHMM-MKL [25] and M-CAP [26]), and 1 score (GERP++) for evolutionary conservation of the affected nucleotide [27].

### Analytical sensitivity and specificity

The analytical sensitivity and specificity of our panel to detect SNVs/indels was calculated using the v3 probe set to evaluate 1624 total variants (1503 SNVs + 121 indels) with allelic frequency ≥ 0.1, following a procedure similar to that previously described [12]. Briefly, 10 immortal lymphoblastoid cell lines, corresponding to 10 individuals whose genomes/exomes had been sequenced by the 1000 Genomes and HapMap projects, were obtained from the Coriell Institute: NA20298 (ASW), NA12872 (CEU), NA18570 (CHB), HG00320 (FIN), HG00110 (GBR), A18960 (JPT), NA19020 (LWK), NA19794 (MXL), HG00740 (PUR) and NA18486 (YRI). Cell lines were cultured according to the protocols provided by Coriell, their DNAs isolated and mixed in equimolecular amounts. An NGS library was prepared, captured using the custom probe and sequenced in 20% of a NextSeq500 MidOutput run (2 × 75 sequencing cycles). Variants were called as described in the previous section and the results compared to those expected according to the genomic information available for these cell lines.

### Interfering highly homologous regions

Regions with < 100% callability at DP20 (i.e. with less than 100% of the target bases covered by ≥20 reads) in > 50% of v3 cases were considered as potential conflictive regions (Additional file 5). Those showing high homology to at least another region of the GRCh38 human reference genome are listed in Additional file 6. Realignment of the NGS results with reference sequences containing only the panel genes affected by these conflictive regions was performed, as previously described for the *PMS2* gene [12], followed by validation, using gene-specific analyses (i.e. Long-Range PCR followed by Sanger sequencing) of putative pathogenic/likely pathogenic SNV and indel variants [28].

### Variant filtering, interpretation, classification and diagnostic yield

Database resources to evaluate gene variants included HGMD professional, OMIM, PubMed, dbSNP, 1000 Genomes Project, ESP, ExAC, and ClinVar. A minor allele frequency (MAF) cut-off of 5% was applied to variants considered as pathogenic (DM) by HGMD or as pathogenic/likely pathogenic by ClinVar, as well as to those variants predicted to create a null allele (nonsense, frameshift causing premature STOP codons, canonical splicing-site disruption, ATG-loss and complete exon deletions/duplications). A MAF cut-off of 1%, as suggested by Shearer et al. [29], was applied to all other variants predicted to affect the sequence/expression of the protein (for protein-coding genes) or of the RNA (for non-coding genes).

Clinical classification of all variants from v1 and v2 cases was performed as described [12]. For v3 cases, only variants that could potentially explain the SNHL phenotype of the probands, based on zygosity of the variant, presence of additional variants on the same gene and mode of inheritance of the audiologic phenotypes associated to the gene, were further considered. After that, variants were clinically classified according to the American College of Medical Genetics and Genomics (ACMG) guidelines as pathogenic (class 5), likely pathogenic (class 4), uncertain significance (class 3), likely benign (class 2) and benign (class 1) [30]. Class 3–5 variants were thoroughly curated searching the literature and databases for clinically relevant data. Class 3–5 variants were reported for tier 1 genes. To reduce the interpretation burden of tier 2 genes, only class 4–5 variants affecting genes whose associated putative phenotype matched the patient’s phenotype were evaluated and reported.

Diagnostic yield (generally described as the likelihood that a test or procedure will provide the information needed to establish a diagnosis) was defined as the percentage of tested patients with pathogenic/likely pathogenic variants capable of explaining their HL phenotype.

### Variant validation

Pathogenic/likely pathogenic variants considered responsible for SNHL were validated by approaches alternative to NGS. SNVs/indels were validated by PCR + Sanger sequencing. For validation and breakpoint identification of the partial *CDH23* duplication (exons 11–15) and the *RDX* exon 2 deletion multiple PCR reactions were performed. *STRC* CNVs were validated with a qPCR assay able to distinguish *STRC* from the *pSTRC* pseudogene [28] as well as by MLPA (MRC-Holland, Amsterdam, The Netherlands; cat.# P64-DIS). Primers used in validation PCRs are described in Additional file 7). Segregation was determined in 8/8 (100%) relatives with available biospecimens.

### Reportable range

Reportable variants had to be supported by ≥4 independent reads, with a total read depth ≥ 10, belonging to genes from Tier 1 (consistently associated) and showing allelic frequencies in the sample ≥ 0.1. Variants considered responsible or likely responsible for the phenotype of the patient additionally needed to have been validated by a method alternative to NGS to be considered reportable. Occasionally, a variant from a Tier 2 gene fulfilling all other criteria could be reportable, as long as the phenotype of the patient was compatible with the phenotype considered in the existing publications supporting the gene-phenotype association of the Tier 2 gene.

### Reference range

Only variants consistent with the mode of inheritance of the auditory phenotypes associated with the gene they affect (for instance, biallelic variants on a gene with a recessive phenotype) were evaluated according to ACMG/AMP guidelines and their resulting clinical classification was reported [30]. For those classified as pathogenic, likely pathogenic or VUS, additional information supporting their clinical classification was provided. Those classified as benign or likely benign were considered to lay within the reference range of results and, thus, no further details about them were provided. The remaining variants (not consistent with the mode of inheritance of the auditory phenotypes associated with the genes they affect) were included in the reports for informative purposes but were not considered responsible for the patient’s phenotype.

### Sample tracking

A series of 6 SNPs on Tier 1 genes, with population MAFs between 0.463 and 0.483, were selected for sample tracking: rs10864198 on *USH2A* (MAF = 0.4531; ExAC); rs7598901 on *ALMS1* (MAF = 0.4736, 1000 Genomes); rs2228557 on *COL4A4* (MAF = 0.4657; ExAC), rs7624750 on *OPA1* (MAF = 0.4683; ExAC), rs734312 on *WFS1* (MAF = 0.4633; ExAC), and rs2438349 on *ADGRV1* (MAF = 0.4830; ExAC). The genotypes identified by the NGS pipeline were compared to those obtained from the corresponding TaqMan qPCR genotyping assays (cat #: C__31803731_10, C__29307975_10, C__11523965_10, C___2715859_10, C___2401729_1 and C__16236492_10, respectively; Applied Biosystems, CA, USA) run on a 7900HT Fast Real-Time PCR System (Applied Biosystems, CA, USA). All samples showed coincident genotypes for all SNPs on both platforms.

### Patient population

Between September 2014 and March 2017, 50 consecutive patients (21 male, 29 female) with syndromic/non-syndromic SNHL were selected after excluding non-genetic causes and causative variants in the *DFNB1 (GJB2/GJB6)*, *OTOF* and *MT-RNR1* loci, considered the most frequent causes of hereditary deafness in Spain [7, 31]. Consent was obtained from patients or their parents. The study was approved by the Comité de Ética de Investigación del Principado de Asturias (research project #75/14). The ages at SNHL onset ranged between 0 to 47 years (median: 12 years). 20 cases (40%) were congenital. To identify syndromic SNHL, a clinical geneticist evaluated the patients. 2/50 patients were diagnosed (pre-test) of Alport and CHARGE syndromes. Other 3 patients presented with potentially syndromic complications, without fulfilling criteria for known syndromes.

## Results

### Panel validation

#### Performance of targeted NGS

Mean coverage of tier 1 genes was 445× for v1, 515× for v2 and 1121× for v3, and 98.87, 99.56 and 99.95% of their target bases were covered by 20 or more reads, respectively (these calculations exclude the *STRC* and *OTOA* genes due to their high homology to other genomic regions). The minimum, average and maximum coverage (average read depth of all target bases of the gene) and callabilities (% of the target bases of the gene with minimum read depths of 10, 20, 50 and 100 reads per each target base of the gene) for every tier 1 and tier 2 gene on samples analysed with OTOgenics v3 is shown in Additional file 5. In v3 cases, regions from tier 1 genes with less than 100% coverage with a minimum of 20 reads (DP20) and specific positions within those regions affected by such limitation were included in each individual patient’s report.

#### Analytical sensitivity and specificity

Prior to its use in the diagnostic setting, the clinical version of the panel (v3) was evaluated for sensitivity and specificity on a genotyped mixture of 10 lymphoblastoid cell lines. 1617/1624 variants with allele frequency ≥ 0.1 were detected (1497/1503 SNVs and 120/121 indels), yielding a sensitivity of 0.9957 (> 99.5%). Additionally, 1,034,047/1034817 true negative positions of the target region were called by the platform as not bearing SNVs or indels, representing a specificity of 0.9992 (> 99.9%).

#### Orthogonal validation of sequencing results

All variants considered responsible for the SNHL phenotypes of the probands (Table 2) were successfully validated by approaches alternative to NGS. These included 25 instances of SNVs or indels (validated by PCR and Sanger sequencing) and 4 CNVs: 1 heterozygous partial duplication of *CDH23* (exons 11–15), 1 homozygous deletion of *RDX* exon 2 (both validated by breakpoint-specific PCR) and 2 homozygous *STRC* whole gene deletions (validated by qPCR and MLPA). Apart from these, 4 samples had heterozygous *STRC* CNVs (3 deletions and 1 duplication) all of which were validated by qPCR and MLPA (Additional file 8). These results indicate that our CNV calling procedure is highly specific.

**Table 2:** Clinical and genetic characteristics of cases with causative mutations

Case ID	Pre-test phenotype	Pre-test suspected inheritance pattern	Time of deafness onset	Gene	Allele variants	Variant zygosity	ACMG^a^ classification^30^	Fulfilled ACMG^a^ pathogenicity criteria^30^	Present in HGMD and/or ClinVar^b^	Gene-associated phenotypes	Inheritance patterns of gene-associated phenotypes	Hidden syndrome
OTO.008	Bilateral non-syndromic sensorineural deafness	AR	Congenital	MYO15A	c.8050 T > C p.(Tyr2684His)	Heterozygous	Likely pathogenic	PM2, PM3, PP1, PP3	Yes	Non-syndromic sensorineural deafness (DFNB3)	AR	No
					c.8968-1G > T	Heterozygous	Pathogenic	PVS1, PM2, PP1	No			
OTO.001	Bilateral non-syndromic sensorineural deafness	AR	Congenital	STRC	Whole-gene deletion	Homozygous	Pathogenic	PVS1, PM2, PM3	Yes	Non-syndromic sensorineural deafness (DFNB16)	AR	No
OTO.033	Bilateral non-syndromic sensorineural deafness	AR	Childhood	STRC	Whole-gene deletion	Homozygous	Pathogenic	PVS1, PM2, PM3	Yes	Non-syndromic sensorineural deafness (DFNB16)	AR	No
OTO.050	Bilateral non-syndromic sensorineural deafness	AD / AR	Childhood	RDX	Exon 2 deletion	Homozygous	Likely pathogenic	PVS1, PM2	No	Non-syndromic sensorineural deafness (DFNB24)	AR	No
OTO.009	Bilateral non-syndromic sensorineural deafness	AR	Congenital	BSND	c.23G > A p.(Arg8Gln)	Homozygous	Likely pathogenic	PM1, PM2, PM5, PP3	No	Non-syndromic sensorineural deafness (DFNB73) / Bartter syndrome type IV	AR	Potential
OTO.006	Bilateral non-syndromic sensorineural deafness	AR	Childhood	SLC26A4	c.412G > T p.(Val138Phe)	Heterozygous	Pathogenic	PS3, PS(PM3), PP3	Yes	Non-syndromic sensorineural deafness (DFNB4) / Pendred syndrome	AR	Potential
					c.1370A > T p.(Asn457Ile)	Heterozygous	Likely pathogenic	PM1, PM2, PM3, PP3	Yes			
OTO.018	Bilateral non-syndromic sensorineural deafness	AR	Congenital	CDH23	c.4488G > C p.(Gln1496His)	Heterozygous	Pathogenic	PS3, PM2, PM3, PP1, PP3	Yes	Non-syndromic sensorineural deafness (DFNB12) / Usher syndrome type 1D	AR	Potential
					Duplication of exons 11–15	Heterozygous	Likely pathogenic	PM2, PM3, PM4	No			
OTO.004	Bilateral non-syndromic sensorineural deafness	AR	Childhood	USH2A	c.11864G > A p.(Trp3955*)	Homozygous	Pathogenic	PVS1, PS(PM3)	Yes	Usher syndrome type 2A	AR	Yes
OTO.005	Bilateral non-syndromic sensorineural deafness	AR	Congenital	USH2A	c.1724G > A p.(Cys575Tyr)	Homozygous	Likely pathogenic	PS(PM3), PM2, PP3	Yes	Usher syndrome type 2A	AR	Yes
OTO.014	Bilateral non-syndromic sensorineural deafness	AR	Congenital	USH2A	c.1724G > A p.(Cys575Tyr)	Heterozygous	Likely pathogenic	PS(PM3), PM2,PP3	Yes	Usher syndrome type 2A	AR	Yes
					c.1841–2A > G	Heterozygous	Pathogenic	PVS1, PS(PM3), PS3, PM2	Yes			
OTO.003	Bilateral non-syndromic sensorineural deafness	AD	Childhood	P2RX2	c.178G > T p.(Val60Leu)	Heterozygous	Likely pathogenic	PS3, PM2, PP1	Yes	Non-syndromic sensorineural deafness (DFNA41)	AD	No
OTO.043	Bilateral non-syndromic sensorineural deafness	AD	Congenital	ACTG1	c.434C > G p.(Ser145Cys)	Heterozygous	Likely pathogenic	PM1, PM2, PP2, PP3	No	Non-syndromic sensorineural deafness (DFNA20/DFNA26) / Baraitser-Winter syndrome type 2	AD	Potential
OTO.023	Bilateral non-syndromic sensorineural deafness	AR	Childhood	ACTG1	c.548G > A p.(Arg183Gln)	Heterozygous	Likely pathogenic	PS2, PP(PM2), PP2, PP3	No	Non-syndromic sensorineural deafness (DFNA20/DFNA26) / Baraitser-Winter syndrome type 2	AD (de novo)	Potential
OTO.041	Bilateral non-syndromic sensorineural deafness	AR	Childhood	ACTG1	c.848 T > C p.(Met283Thr)	Heterozygous	Likely pathogenic	PS2, PM2, PP2	No	Non-syndromic sensorineural deafness (DFNA20/DFNA26) / Baraitser-Winter syndrome type 2	AD (de novo)	Potential
OTO.011	Unilateral non-syndromic sensorineural deafness	AD	Childhood	MITF	c.909G > A p.(Thr303Thr)	Heterozygous	Likely pathogenic	PS3, PM2, PP1	Yes	Waardenburg syndrome type 2A	AD	Yes
OTO.051	Bilateral non-syndromic sensorineural deafness	AR	Congenital	SOX10	c.135_154del p.(Ser45Argfs*15)	Heterozygous	Likely pathogenic	PVS1, PM2, PP3	No	Waardenburg syndrome type 2E	AD	Yes
OTO.019	Bilateral non-syndromic sensorineural deafness	AR	Congenital	GATA3	c.1018A > C p.(Asn340His)	Heterozygous	Likely pathogenic	PM1, PM2, PM6, PP2, PP3	No	Barakat syndrome	AD (de novo)	Yes
OTO.010	CHARGE syndrome	AD	Congenital	CHD7	c.235A > T p.(Lys79*)	Heterozygous	Pathogenic	PVS1, PM2, PP4	No	CHARGE syndrome	AD	No
OTO.015	Bilateral non-syndromic sensorineural deafness	AR	Childhood	POU3F4	c.692C > T p.(Thr231Ile)	Hemizygous (male)	Likely pathogenic	PM1, PM2, PP2, PP3	No	Non-syndromic sensorineural deafness (DFNX2/DFN3)	XR	No
OTO.016	Bilateral non-syndromic sensorineural deafness	AD	Childhood	PRPS1	c.826C > T p.(Pro276Ser)	Hemizygous (male)	Likely pathogenic	PM1, PM2, PP2, PP3	No	Non-syndromic sensorineural deafness (DFNX1)	XD	No
OTO.007	Alport syndrome	AR	Childhood	COL4A5	c.3525_3529dup p.(Pro1177Leufs*124)	Hemizygous (male)	Pathogenic	PVS1, PM2, PM6	No	Alport syndrome	XD (de novo)	No

GenBank Accession and version numbers of the genes listed in the table: ACTG1 (NM_001614.3), BSND (NM_057176.2), CDH23 (NM_022124.5), CHD7 (NM_017780.3), COL4A5 (NM_000495.4), GATA3 (NM_001002295.1), MITF (NM_000248.3), MYO15A (NM_016239.3), P2RX2 (NM_174873.2), POU3F4 (NM_000307.4), PRPS1 (NM_002764.3), RDX (NM_002906.3), SLC26A4 (NM_000441.1), SOX10 (NM_006941.3), STRC (NM_153700.2), USH2A (NM_206933.2)

^a^American College of Medical Genetics and Genomics

^b^Yes: variants present in HGMD and/or Clinvar at the moment of clinical interpretation of the case; No: variants absent from both HGMD and ClinVar at the moment of clinical interpretation of the case

#### Performance at interfering highly homologous regions

Genomic regions with high sequence homology cause misalignment of sequencing data and represent a major challenge for short-read NGS technologies. Out of the 199 genes included in the v3 panel, *STRC*, *OTOA*, *ESPN* and *KCNE1* contain a total of 22 interfering highly homologous regions (as defined in the Methods section), most of which overlap with those previously identified by Mandelker et al. [32] (Additional file 6). To avoid missing clinically relevant variants present in those target regions, the panel NGS reads from all samples were realigned to reference sequences containing only the *STRC*, *OTOA*, *ESPN* and *KCNE1* loci, as previously described by us for the *PMS2* gene in a cancer panel [12]. This approach revealed that all samples might potentially carry a pathogenic variant in the *STRC* gene: c.4057C > T, p.Gln1353* (coincident with the reference sequence for exon 20 from the *pSTRC* pseudogene). To unequivocally discriminate the origin of this variant, LR-PCR specific for the *STRC* gene followed by Sanger sequencing was performed as described [28]. This approach discarded the potential genic origin of the variant in 28/50 samples; not enough DNA was available from 1 sample and no LR-PCR product was obtained from 21. Of note, the average genomic DNA Integrity Number (DIN) of the 28 samples with successful LR-PCR was significantly higher than that of the remaining 21 samples (8.71 vs 7.21; *p*-value = 1.8 × 10^− 4^; Student’s T test), suggesting that DNA degradation precluded LR-PCR. Alternative approaches would be needed to discard or confirm the genic origin of the variant in those 21 samples.

### Analysis of causative variants and diagnostic yield

Of 50 cases with severe-to-profound SNHL not caused by *GJB2/GJB6*, *OTOF* or *MT-RNR1* mutations, a genetic justification for their HL phenotype was found in 21 (42%) after identifying 31 pathogenic/likely pathogenic variants in 16 genes: *ACTG1*, *BSND*, *CDH23*, *CHD7*, *COL4A5*, *GATA3*, *MITF*, *MYO15A*, *P2RX2*, *POU3F4*, *PRPS1*, *RDX, SLC26A4*, *SOX10*, *STRC* and *USH2A* (Tables 2 and 3). Three more cases had recessive variants of uncertain significance in homozygosis (affecting the *LOXHD1* and *SLC26A4* genes) or in hemizygosis (affecting the *OTOA* gene: 1 SNV + heterozygous whole-gene *OTOA* deletion), which were suspicious of pathogenicity, but did not fulfill ACMG criteria and, thus, were not counted nor reported as positives (Table 4). Had they been counted, the diagnostic yield would have been 48% (24/50).

**Table 3:** Clinical characteristics of cases without causative mutations

Case ID	Phenotype	Suspected inheritance pattern	Time of deafness onset
OTO.017	Bilateral non-syndromic sensorineural deafness	AR	Congenital
OTO.021	Bilateral sensorineural deafness. Nystagmus, strabismus, delay in psychomotor development and autism spectrum disorder	AR	Congenital
OTO.024	Bilateral non-syndromic sensorineural deafness	AR	Childhood
OTO.025	Bilateral non-syndromic sensorineural deafness	AR	Childhood
OTO.026	Unilateral non-syndromic sensorineural deafness	AR	Childhood
OTO.027	Bilateral non-syndromic sensorineural deafness	AR	Congenital
OTO.028	Bilateral non-syndromic sensorineural deafness	AR	Childhood
OTO.029	Unilateral non-syndromic sensorineural deafness	AR	Congenital
OTO.030	Unilateral sensorineural deafness. Connective tissue problems, digestive problems, urinary reflux and knee hypermobility	AR	Childhood
OTO.032	Unilateral non-syndromic sensorineural deafness	AR	Congenital
OTO.034	Bilateral non-syndromic sensorineural deafness	AR	Childhood
OTO.035	Bilateral non-syndromic sensorineural deafness	AR	Childhood
OTO.038	Bilateral non-syndromic sensorineural deafness	AR	Unknown
OTO.040	Bilateral non-syndromic sensorineural deafness	AR	Childhood
OTO.042	Bilateral non-syndromic sensorineural deafness	AR	Congenital
OTO.044	Bilateral non-syndromic sensorineural deafness	AR	Congenital
OTO.045	Bilateral non-syndromic sensorineural deafness	AR	Congenital
OTO.046	Bilateral non-syndromic sensorineural deafness	AR	Childhood
OTO.049	Bilateral non-syndromic sensorineural deafness	AR	Adulthood
OTO.052	Bilateral sensorineural deafness. Lobe of the auricular pavilion with grooves. Polysyndactyly in hands and feet. Hypospadias	AR	Congenital
OTO.053	Bilateral non-syndromic sensorineural deafness	AR	Childhood
OTO.036	Bilateral non-syndromic sensorineural deafness	AR/AD	Childhood
OTO.039	Bilateral non-syndromic sensorineural deafness	AR/AD	Adulthood
OTO.020	Bilateral non-syndromic sensorineural deafness	AD	Childhood
OTO.022	Bilateral non-syndromic sensorineural deafness	AD	Congenital
OTO.031	Bilateral non-syndromic sensorineural deafness	AD	Childhood
OTO.037	Bilateral non-syndromic sensorineural deafness	AD	Adulthood
OTO.047	Bilateral non-syndromic sensorineural deafness	AD	Childhood
OTO.048	Bilateral non-syndromic sensorineural deafness	AD	Childhood

**Table 4:** Clinical and genetic characteristics of cases with suspicious VUS^#^

Case ID	Pre-test phenotype	Pre-test suspected inheritance pattern	Time of deafness onset	Gene	Allele variants	Variant zygosity	ACMG^¥^ classification^30^	Fulfilled ACMG^¥^ pathogenicity criteria^30^	Gene-associated phenotype	Inheritance patterns of gene-associated phenotypes	Hidden syndrome
OTO.028	Bilateral non-syndromic sensorineural deafness	AR	Childhood	*OTOA*	Whole-gene deletion	Heterozygous	Pathogenic (likely pathogenic overriden to pathogenic)	PVS1, PM3	Non-syndromic sensorineural deafness (DFNB22)	AR	No
					c.1282G > T (p.Val428Phe)	Hemizygous	VUS^#^	PM2, PM3, PP2			
OTO.044	Bilateral non-syndromic sensorineural deafness	AR	Congenital	*LOXHD1*	c.3571A > G (p.Thr1191Ala)	Homozygous	VUS^#^	PM2, PP3	Non-syndromic sensorineural deafness (DFNB77)	AR	No
OTO.045	Bilateral non-syndromic sensorineural deafness	AR	Congenital	*SLC26A4*	c.695 T > G (p.Leu232Arg)	Homozygous	VUS^#^	PM1, PM2, PP3	Non-syndromic sensorineural deafness (DFNB4)/ Pendred syndrome	AR	Potential

^#^Variant/s of unknown significance

^¥^American College of Medical Genetics and Genomics

In our cohort, 47.6% (10/21) of the unambiguously molecularly diagnosed patients had autosomal recessive (AR) inheritance patterns, 38.1% (8/21) autosomal dominant (AD), and 14.3% (3/21) were X-linked (Table 2). The molecular basis of deafness was found in 44.4% (20/45) of the cases with symmetric SNHL, whereas only 1 of 5 cases with asymmetric SNHL was genetically diagnosed (Waardenburg syndrome caused by a *MITF* mutation) (Tables 2 and 3).

The most common SNHL causative genes in our pre-screened population were *ACTG1* (3 patients), *USH2A* (3 patients) and *STRC* (2 patients). Interestingly, 2 of 3 pathogenic variants in *ACTG1* were de novo, as well as 1 *GATA3* and 1 *COL4A5* pathogenic variants.

CNV analysis identified causative variants in 4 of the 21 molecularly diagnosed patients (19%): 2 with a homozygous complete *STRC* deletion, 1 with a previously unreported partial *CDH23* duplication (exons 11–15) in compound heterozygosity with a second pathogenic variant (missense), and 1 with a homozygous *RDX* exon 2 deletion. One of the patients with a homozygous causative *STRC* deletion was also a carrier of a heterozygous substitution in *TECTA*, previously reported in the literature as a dominant pathogenic variant (c.3107G > A; p.Cys1036Tyr) [33]. However, revaluation of this variant according to ACMG guidelines reclassified it as a variant of uncertain significance (it only fulfilled ACMG pathogenicity criteria PP1 and PP3).

**Fig. 1:**
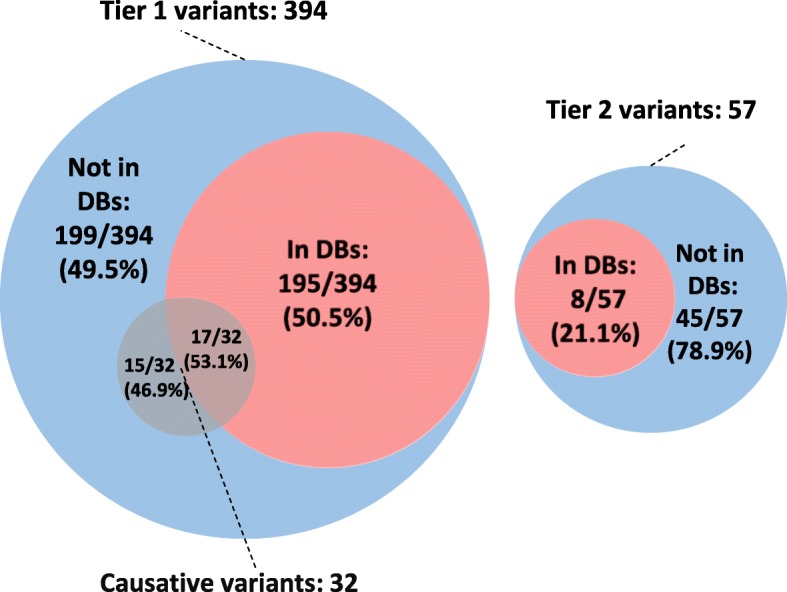
Presence/absence of total and causative variants in databases. Circles represent total numbers of tier 1 (left) and tier 2 (right) variants (not to scale), their presence in the HGMD professional and/or ClinVar databases (in DBs) or their absence from both databases (not in DBs) at the moment of case evaluation, and the distribution of the 32 variants considered causative of HL within these categories

In total, 451 variants, of which 406 were unique, in 121 distinct genes were identified in the full cohort of 50 patients: 394 variants in 97 genes were identified in tier 1. Tier 2 added 57 variants that contributed to the overall interpretation burden. No tier 2 variant was considered responsible for the SNHL phenotype (Fig. 1).

**Fig. 2:**
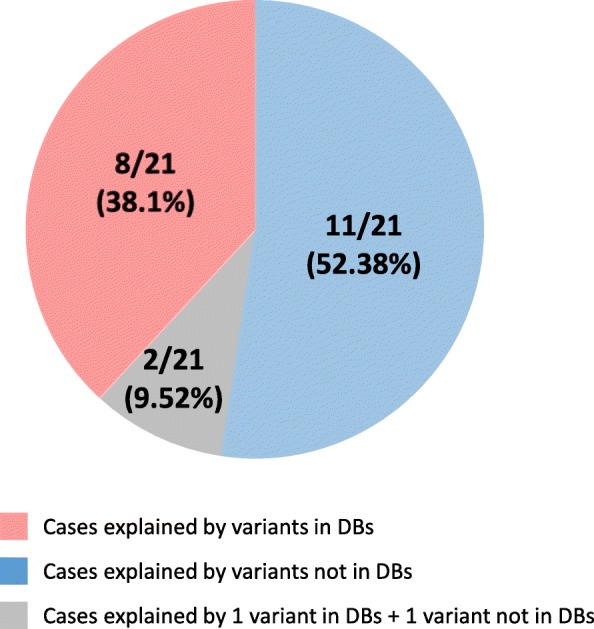
Genetically diagnosed cases explained by variants present in and absent from databases. Sectors represent the percentage of genetically diagnosed cases explained by variants present in (in DBs) or absent from (not in DBs) the HGMD professional and/or ClinVar databases at the moment of case evaluation

199/394 (50.5%) and 45/57 (78.9%) of the identified tier 1 and tier 2 variants, respectively, were absent from the databases (HGMD professional and ClinVar). Fifteen of them (all from tier 1), were classified as pathogenic or likely pathogenic and responsible for the SNHL phenotype of the patient (Fig. 1). Globally, those 15 variants were considered responsible for the SNHL phenotype in 13 cases. As a result, 61.9% of the genetically diagnosed cases (13/21) were explained by variants not described in the databases (Fig. 2 and Table 2). Moreover, of 25 non-redundant variants classified as pathogenic (DM) by HGMD for hearing-related phenotypes, after looking for plausible published support in the literature, solid evidence could only be found for 13 of them (52%) (Additional file 9). Of the 12 variants considered DM by HGMD but, in our view, without enough supporting evidence, 3 (25%) were also considered as Pathogenic/Likely pathogenic by at least one ClinVar submitter (Additional file 9). To deal with these limitations, an average of 40 min of expert review was dedicated per variant. With an average of 9 variants per case, this represents 360 min (6 h) per case. These results highlight the importance of manual interpretation and curation for clinical classification of variants, even for those considered as (potentially) disease causing by reputable databases.

### Increase of clinical sensitivity by analysis of syndromic genes on apparently non-syndromic SNHL

28.6% of the genetically diagnosed cases (6/21) had a previously unrecognized syndrome: Barakat (1 patient), Usher type 2A (3 patients) and Waardenburg (2 patients) (Table 2). These unexpected syndromic findings not only increased the diagnostic yield, but they provided diagnostics of utmost clinical relevance. Additionally, 6 patients carried pathogenic variants in genes associated with syndromic and non-syndromic conditions (Table 2): 1 had variants associated with Pendred syndrome and DFNB4, 1 with Bartter syndrome type IV and DFNB73, 1 with Usher syndrome 1D and DFNB12 and 3 with Baraitser-Winter type 2 and DFNA20/DFNA26. These patients will need close follow-up in case syndromic features develop.

## Discussion

Hearing loss is one of the most genetically heterogeneous disorders known. 60% of cases are believed to be of genetic origin and 30% of them syndromic [34]. Due to its high diagnostic yield [35, 36], the newest ACMG guidelines include NGS testing in the standard SNHL diagnostic algorithm [1], whereas the use of non-genetic tests should be considered case-by-case, usually as a complement to genetic testing. However, except for the preeminent relevance of *GJB2* mutations, little is known about the frequency of SNHL variants in Europeans [9, 37].

Our results contribute to define the mutation spectra in the Spanish population, underlining the SNHL genetic heterogeneity, as the causative variants of 21 patients affected 16 different genes. The genes most commonly altered in our pre-screened population were *ACTG1* (*n* = 3), *USH2A* (n = 3) and *STRC* (n = 3). Although variants in *USH2A* and *STRC* are often reported as common causes of SNHL [28, 37–40], the identification of *ACTG1* as the most frequent causative gene in our cohort is surprising.

*ACTG1* variants are responsible for DFNA20/DFNA26 and type 2 Baraitser-Winter syndrome. None of our 3 cases has syndromic features to date, and all of them had early-onset profound SNHL, expanding the phenotypic spectrum of *ACTG1*, usually associated to post-lingual and progressive SNHL [41, 42], to prelocutive SNHL. Since none of the 3 causative variants had been described and 2 of them were de novo, a targeted hot-spot mutation assay or an AR oriented gene panel (the 2 de novo mutations took place in patients without familial background, simulating a recessive pattern) would have missed them. Therefore, the prevalence of *ACTG1* pathogenic variants could be higher, and its expression pattern more variable, than previously thought [43].

Our 38.1% rate of syndromic SNHL (8/21, including 2 syndromes diagnosed before and 6 after NGS genetic testing) is within expected rates. In contrast, our 38% incidence of AD and 14% of X-linked SNHL are higher than expected [44]. This might reflect the consequences of pre-screening, which excluded the most common AR (*GJB2/GJB6* and *OTOF*) and mitochondrial (*MT-RNR1*) mutations [2, 31, 45]. However, since in our patients 50% (4/8) of causative dominant variants were de novo (2 in *ACTG1*, 1 in *GATA3*; and 1 in *COL4A5*, Table 2) it might also be the consequence of using an unbiased NGS panel, able to identify unexpected de novo variants. Despite the limited size of our cohort, our de novo detection rate is strikingly similar to that reported recently in whole-exome sequencing (WES) studies for different clinical indications (37–68%) [35, 46].

A technical difficulty encountered for the implementation of a clinical-grade test was the presence of highly homologous pseudogene background for some of the target genes included in the panel (Additional file 6), especially *STRC* and *OTOA*. The measures proposed to deal with this problem (gene-restrictive realignment of sequencing results and validation of putative causative variants by gene-specific methods) should reduce misdiagnosis. Moreover, the *STRC* gene, one of the largest contributors to AR SNHL [28, 47, 48], is also a common site for large deletions [28], and CNVs can be refractory to general NGS approaches. As displayed in our population, where 19% of cases (4/21) were justified by CNVs, large genetic rearrangements are increasingly recognized as a common cause of genetic hearing loss, accounting for 13–19% of all causative variants [5, 48–50]. Therefore, CNV analysis should be a requirement for all patients undergoing genetic testing for SNHL. In this regard, our 100% validation rate of NGS-detected *STRC* CNVs by qPCR and MLPA is encouraging (Additional file 8).

Diagnostic rates of up to 60% are expected in patients with suspected AR congenital deafness. This percentage strongly declines for AD hearing loss, especially with the increase in the age of onset [5, 48]. In our series, as in most of published studies [5, 48, 51], prior to comprehensive genetic testing, patients were prescreened for common deafness mutations (in our cohort, *GJB2/GJB6*, *OTOF* and *MT-RNR1*, selected for their high prevalence in Spain [7, 31]). Mutations in the *GJB2* gene are among the most frequent causes for congenital hearing loss. The prevalence of its biallelic pathogenic mutations among non-syndromic SNHL cases ranges geographically from 0% to over 50% [3, 52–54]. Recent analyses show a worldwide and European prevalence of around 13%, increasing in < 5 year-old patients [2, 3]. In our laboratory, *GJB2/GJB6*, *OTOF* and *MT-RNR1* prescreening of 180 patients identified the cause of deafness in 34 (18.9%) (unpublished results). This figure, combined with the 42–48% diagnostic rate of our panel in pre-screened patients (48% considering as causative the highly suspicious variants of Table 4), allows us to estimate that combining prescreening with our panel will lead to a diagnosis in about 53–58% of patients.

Our 42–48% detection rate is slightly higher than the average reported with NGS-panels: 41% (10–83%) for a mix of pre-screened and not pre-screened patients [5, 9, 28, 48, 51]. Proper target region coverage and bioinformatics approaches shouldn’t be underestimated for maximizing clinical sensitivity. Additionally, the inclusion of syndromic genes, revealing ‘hidden syndromes’, increased the diagnostic yield. The 6 a priori clinically unrecognized syndromes in our cohort diagnosed after genetic testing (Table 2), representing 28.6% of the genetically diagnosed cases, are a proof of concept of how NGS is changing medicine. In fact, undiagnosed syndromes in families with apparently non-syndromic SNHL are increasingly reported [55–58], expanding the phenotypes associated with SNHL-syndromes [35]. Moreover, 6 patients in our series had pathogenic variations in genes associated with both syndromic and non-syndromic HL: *CDH23* (Usher syndrome 1D and DFNB12), *ACTG1* (Baraitser-Winter syndrome type 2 and DFNA20/DFNA26), *BSND* (Bartter syndrome type IV and DNFB73) or *SLC26A4* (Pendred syndrome and DFNB4) (Table 2). Close follow-up of these patients is mandatory, since syndromic features may develop.

The clinical interpretation of genomic findings is a cornerstone of NGS diagnostic pipelines. Beyond deafness, a recent study indicated that as many as 30% of all disease-causing genetic variants cited in the literature may have been misinterpreted [59]. In our cohort, manual interpretation of variants required an average of 6 h/case, dedicated to in-depth review of the databases and scientific literature, under the perspective of the patient’s phenotype and family history, which is imperative for accurate variant interpretation [11, 60].

To date, several studies using NGS for genetic diagnosis of deafness have been published, involving either gene panels or WES [5, 61]. When WES is ordered, sequenced regions not only include genes of interest (“targeted disease-specific panels” such as the one presented in this paper), but also all exons of all genes in the genome. Although WES avoids the need for specific gene panel enrichment, a literature-based selection of the genes involved in the pathology is anyway required for results intepretation. WES increases the requirements for sequencing resources, complicates the analysis and normally provides insufficient coverage of key target regions [29]. Moreover, WES carries increased chance of secondary findings (variants identified in genes unrelated to the primary medical reason for testing [62])*,* which introduce noise into the genetic counseling procedure. A comparison of a disease-focused panel versus WES for inherited eye diseases found improved accuracy and performance of the disease-specific panel, a finding that can be translated to hearing loss panels [63]. For these reasons, disease-focused genetic tests have become the standard when evaluating hearing loss [64]. However, WES does have an advantage: the ability to identify alterations in genes not definitely associated with the disease yet. To minimize this disadvantage, a tiered approach was implemented: tier 1 includes all genes consistently associated with SNHL, whereas tier 2 includes genes without sufficient clinical validity to be included in clinical testing. Non-systematic reporting of tier 2 genes reduces the uncertainty and simplifies the genetic counseling procedure. However, meanwhile, it facilitates fast pipeline incorporation of clinically validated genes, as soon as confirmatory discoveries are published.

## Conclusions

Our results underscore the importance of a comprehensive approach with careful gene selection to the genetic diagnosis of SNHL. Here, we contribute to show that, with the right methodology, NGS can be transferred to the clinical practice, boosting the yield of SNHL genetic diagnosis to 50–60% (including *GJB2/GJB6* alterations), improving diagnostic/prognostic accuracy, refining genetic and reproductive counseling and revealing clinically relevant undiagnosed syndromes. Lowering cost and increasing quality of WES and whole-genome sequencing (WGS) will probably prompt substitution of physical gene panels by non-targeted approaches. However, WES/WGS results are likely to be filtered through *in-silico* gene panels, based on a meticulously curated gene selection, such as the gene-set of the current panel. Thus, the methodology implemented on the present study is expected to be useful in the years to come. Since comprehensive genetic testing using NGS should be the standard of care for genetic evaluation of patients with SNHL, hereditary deafness should become a paradigm on the raising field of precision medicine. In this context, we expect that the use of the current platform, or others developed on the knowledge presented herein, will help to bring to the clinical arena the advantages of predictive and preventive SNHL genetic testing.

## Additional files

Additional file 1: List of phenotypes potentially related to hearing defects used as keywords for initial query on HGMD. (XLSX 16 kb)

Additional file 2: Tier 1 and tier 2 genes included in v1 of the panel. (XLSX 13 kb)

Additional file 3: Tier 1 and tier 2 genes included in v2 of the panel. (XLSX 13 kb)

Additional file 4: Cases analyzed with each panel version. (XLSX 10 kb)

Additional file 5: Coverages and callabilities for every tier 1 and tier 2 gene on v3 samples. (XLSX 31 kb)

Additional file 6: Highly homologous conflictive regions. (XLSX 11 kb)

Additional file 7: Primers used to validate causative (pathogenic and likely pathogenic) variants. (XLSX 13 kb)

Additional file 8: Samples with STRC CNVs. (XLSX 9 kb)

Additional file 9: Variants identified in our cohort classified as pathogenic (DM) by HGMD for hearing-related phenotypes. (XLSX 11 kb)

## Abbreviations

ACMG: Americal college of medical genetics and genomics
AD: Autosomal dominant
AR: Autosomal recessive
CNVs: Copy number variations
DIN: DNA integrity number
HL: Hearing loss
NGS: Next-generation sequencing
SNHL: Sensorineural hearing loss
SNVs: Single nucleotide variants
WES: Whole-exome sequencing
WGS: Whole-genome sequencing
